# The role of natural health products (NHPs) in dietetic practice: results from a survey of Canadian dietitians

**DOI:** 10.1186/1472-6882-13-156

**Published:** 2013-07-03

**Authors:** Kristine Hirschkorn, Rishma Walji, Heather Boon

**Affiliations:** 1 Ontario Health Human Resources Research Network, University of Ottawa - Institute of Population Health, 1 Stewart St., room 227, Ottawa, Ontario, Canada K1H 8M5; 2 Canadian College of Naturopathic Medicine, 1255 Sheppard Avenue East, Toronto, Ontario, Canada M2K 1E2; 3 Leslie Dan Faculty of Pharmacy, University of Toronto, 144 College Street, Toronto, Ontario, Canada M5S 3M2

**Keywords:** Dietitians, Professional roles and responsibilities, Natural health products, Dietary supplements, Nutritional supplements, Functional foods, Nutraceuticals, Herbal preparations

## Abstract

**Background:**

Registered dietitians (RDs) play a key role in disseminating information about nutrition and intervening in nutrition-related disorders in the Canadian context. Natural health products (NHPs) are increasingly associated with nutrition in patient and health professional discussions. For this study, NHPs were divided into three categories: nutritional supplements (NS); functional foods/nutraceuticals (FF/N); and herbal preparations (HP). The objective was to explore RDs’ perceptions about their professional roles and responsibilities with respect to three categories of natural health products (NHPs).

**Methods:**

This research consisted of an on-line survey of registered dietitians (RDs) in Ontario.

Surveys were distributed electronically to all practicing RDs in Ontario by the College of Dietitians of Ontario. There were 558 survey respondents, a response rate of 20%.

**Results:**

The vast majority of RDs reported being consulted by clients about all product categories (98% for NS; 94% for FF/N; 91% for HP), with RDs receiving the most frequent questions about NS and the least frequent about HP. 74% of RDs believed that NS are included within the current scope of practice, compared to 59% for FF/N and 14% for HP. Even higher numbers believed that these products should be included: 97% for NS, 91% for FF/N and 47% for HP. RDs who report personally ingesting FF/N and HP were significantly more likely to report that these products should be in the dietetic scope of practice. In contrast, RDs who provide one-on-one counselling services or group-level counselling/workshops were significantly less likely to believe HP should be in the dietetic scope of practice.

**Conclusions:**

Opinions of RDs indicated that NS and FF/N (and possibly HP) fall within, or should fall within, RDs’ scope of practice. Opportunity exists for RDs to undertake a professional role with respect to NHPs. Policy clarification regarding RD roles is needed.

## Background

Dietitians play a key role in disseminating information about nutrition and intervening in nutrition-related disorders in the Canadian context. Despite provincial variation in regulations, the scope of practice of dietitians in Canada is generally defined as follows: “The practice of dietetics is the assessment of nutrition and nutritional conditions and the treatment and prevention of nutrition related disorders by nutritional means” [1].

National guidelines for the use of dietary or nutritional supplements for specific population groups exist (e.g., folic acid for women capable of becoming pregnant) [2,3]. While these guidelines are published for the general public, dietitians play an important part in patient education. According to the former American Dietetic Association (now the Academy of Nutrition and Dietetics), “[t]he expertise of dietetics practitioners is needed to help educate consumers on safe and appropriate selection and use of dietary supplements, including nutrient supplements” (p. 2074) [4]. The Association also outlined a position for functional foods, in which they argue that “defining and utilizing functional foods is an important component of [dietitians’] practice. They are consumers’ bridge between evidence-based research and optimal health” (p. 736) [5].

For these reasons, it is important to know more about the dietitian perspective on these products, which are included in Health Canada’s definition of natural health products (NHPs) [6]. Functional foods and nutraceuticals are typically regulated as foods under Canada's *Food and Drugs Act*, while some related products, including nutritional supplements and herbal products, are currently regulated as natural health products (NHPs) under the same Act. The legal definition of NHPs is: natural source “substances which are manufactured, sold or represented for use in: i) the diagnosis, treatment, mitigation or prevention of a disease, disorder, or abnormal physical state or its symptoms in humans; ii) restoring or correcting organic functions in humans; or iii) maintaining or promoting health or otherwise modifying organic function in humans” (p. 1536) [6]. All products covered by the Regulations must be in dosage forms (i.e., bulk herbs are not included) and must have a wide margin of safety.

NHPs are widely available across Canada, and consumption of NHPs by the Canadian population – along with visits to practitioners who recommend their use – has continued to rise [7]. Notwithstanding these trends, there has been relatively little discussion among members of the dietetic profession about what professional responsibilities dietitians have (or should have) with respect to these products.

A limited number of studies exist that address this topic [8-12]. The one telephone survey of 151 dietitians in Canada indicated that participants overwhelmingly (n = 122, 81%) felt that dietitians were the most appropriate professionals to recommend functional foods, but held mixed views of the appropriateness of having dietitians recommend nutraceuticals [12]. However, respondents across all areas of practice believed that it is extremely important for dietitians to become knowledgeable about nutraceuticals and functional foods [12].

In U.S. studies, dietitians’ knowledge about nutritional supplements and functional foods was reportedly higher than knowledge about herbal products [8], and overall higher than other practitioner groups [9]. There was also a perceived need for more training/knowledge for American dietitians [8,10], which is not surprising given the high frequency of questions that interns/directors reported receiving from clients about vitamins/minerals and herbal products in the five years preceding the study [10]. It was also reported that the majority of Dutch dietitians in counselling advised about the use of functional foods [11]. Overall, however, this literature is largely speculative about the potential implications and responsibilities for dietitians or focuses on ethical considerations around the sale of NHPs [13-17]. Few guidelines exist for products other than vitamins/minerals.

One notable exception is the set of guidelines published by the (former) American Dietetic Association [17] that were developed in response to the increasing expectation that dietitians provide recommendations with respect to dietary supplements, the U.S. regulatory category that includes nutritional supplements and herbal products [18]. The basis of these guidelines is “Ask, evaluate, educate & document”. The authors of this paper argue that “given our education and training in diet and nutrition, our profession is uniquely positioned to meet this need” (p. 1158). This expectation is, however, coupled with an overall lack of familiarity on the part of most dietitians with this area of practice or with the legal and ethical issues that accompany it [19]. While the content of these recommendations point to some possible directions for Canadian dietitians, given the different regulatory environment here, further research and dialogue is required before such guidelines can arise in the Canadian context. A similar gap for the pharmacy profession resulted in the development of core competencies for Canadian pharmacy students [20], but the equivalent does not exist for Canadian registered dietitians (RDs).

It remains unclear how Canadian and Ontario dietitians are negotiating the use of, or demand for, various NHPs in their practices. The purpose of the research undertaken here was to explore RDs’ practice behaviors and perceptions about their professional activities with respect to NHPs in an effort to articulate existing or potential professional roles and responsibilities. Specifically, “roles” refer to activities or services that individual RDs may competently perform or provide as part of their professional scope of practice (e.g., as an area of specialization), whereas “responsibilities” refer to activities or services that all RDs across service areas or RDs within a particular service area are required to demonstrate the capacity to perform, i.e., for which entry-to-practice or essential competencies have been established [21]. The research questions were: What are registered dietitians (RDs) saying and doing about NHPs in practice? And ultimately, what are the roles and responsibilities of RDs with respect to NHPs?

In the results that follow, three main categories of products are addressed: nutritional supplements (NS); functional foods/nutraceuticals (FF/N); and herbal preparations (HP). Some of the products falling in these categories overlap with foods, i.e., are not strictly NHPs. For example, functional foods may be considered primarily ‘food’ products and consequently fall under Canadian federal ‘food’ rather than NHP regulations. Nonetheless, these product groups were chosen because they largely represent the way the RDs who were interviewed in a pilot study talked about the products and their intentions for their use.

Nutritional supplements (or NS) refer to those products that are intended to meet basic nutritional needs. A small number of questions in the survey asked RDs about NS that exceed the Recommended Dietary Allowance (RDA) / Adequate Intake (AI). Examples of NS include: vitamin/mineral supplements, protein powders, and meal replacements.

The intention for use of functional foods/nutraceuticals (or FF/N), in contrast, is medicinal – i.e., their use is beyond a basic nutrition function. This means, for example, that the medicinal effect is for reducing risk or providing protection against chronic disease, and/or providing physiologic benefits. The distinction between functional foods and nutraceuticals is their formulation: functional foods generally appear food-like, whereas nutraceuticals are typically isolates from foods. Examples of nutraceuticals include: amino acids, and fish oils.

Finally, the intention for use of herbal preparations (or HP) is also medicinal; however, the source of HP is generally not food or food products. They may be in various forms such as loose herbs, pills/capsules, teas or other liquids that maintain original molecular structure (i.e. not isolates). Some examples include: echinacea, ginseng, St. John's wort, garlic capsules, other single herbs or formulations containing these and other herbs.

## Methods

An on-line survey was distributed to all registered dietitians (RDs) in Ontario (N = 2,780) in 2007. Prior to administering the survey, the questionnaire was reviewed by eight colleagues (including three methods experts) and four dietitian leaders, and was pre-tested with 13 RDs in provinces other than Ontario. Following pre-testing, minor modifications were made, including correction of spelling errors, change of question order, changes of some response categories and deletion of some questions. Finally, an on-line survey was distributed to all registered dietitians (RDs) in Ontario (N = 2,780) by email invitation through the College of Dietitians of Ontario (CDO).

Ethics approval for this study was attained through the Ethics Review Office, University of Toronto.

The survey questionnaire was developed to examine perceptions and practices of dietitians with regard to NHPs. The survey was comprised of four main topic areas. The first section included demographic questions relating to the age, gender, educational background, and practice characteristics of each dietitian. The second section comprised questions designed to assess the demand for dietitians’ services, as well as their behaviours (and for a subset, their counselling practice patterns) with respect to the three product groups (NS, FF/N, HP). The third section was dedicated to examining the education level of dietitians for each product group. The final section included questions about dietitians’ perspectives on whether NHPs should be included in their scope of practice, again by product group.

Data were collected into an excel table, then migrated to SPSS where they were cleaned, recoded, and analyzed. In addition to descriptive statistics, bivariate relationships were explored with cross-tabs using the chi-square statistic, and a logistic regression model was fit.

## Results and discussion

Results are presented in four main categories: response rate and respondent demographics, dietitians and NHPs, demand for NHPs in dietetic services, and views about inclusion of NHPs in dietetic scope of practice. The results reported here do not include an overview of counselling practice patterns.

Note that throughout the presentation of the results, the survey respondents will simply be referred to as ‘Registered Dietitians’ (RDs).

### Response rate and respondent demographics

Based on August 2007 statistics provided by the College of Dietitians of Ontario (CDO), the calculated survey response rate is 20% (N = 558). For the logistic regression analysis, after excluding missing cases, N = 475 for the FF/N model and N = 472 for the HP model.

In Table 1, the primary employment settings of RDs who responded to the survey are compared with those of the full population of RDs in Ontario. RDs from the full population may have identified more than one primary employment setting. RDs working in hospitals were by far the most numerous group in practice and in our study. For hospitals and other primary employment settings with substantial numbers of RDs (e.g., community health centre/agency/clinic, and public health department/unit), the percentage of RDs in our study was comparable. RDs working in other settings were in some cases underrepresented (e.g., chronic care/long term care residence, private practice and counselling), although these RDs constituted a smaller proportion of the practicing population.

**Table 1:** Primary employment settings of RDs

**Primary employment setting**	**RD Survey Respondents N (%)**	Full population of RDs in ON* N (%)**
Hospital	245 (45.1)	1120 (40.3%)
Community health centre/agency/clinic	51 (9.4)	227 (8.2)
Public health department/unit	50 (9.2)	250 (9.0)
Chronic care/LTC residence	38 (7.0)	404 (14.5)
Private practice & counselling	23 (4.2)	289 (10.4)
Government	16 (2.9)	188 (6.8)
Food & pharmaceutical industry	14 (2.6)	115 (4.1)
Professional services	11 (2.0)	60 (2.2)
CCAC/ home care program /agency	11 (2.0)	148 (5.3)
University/ Community college	11 (2.0)	155 (5.6)
Business	10 (1.8)	178 (6.4)
Home for the aged	10 (1.8)	107 (3.8)
NGO/not-for-profit organization	8 (1.5)	61 (2.3)
Rehabilitation centre	8 (1.5)	53 (1.9)
Other	37 (6.8)	112 (4.0)
Total	558 (~100%)	2780 (124.8%)

*Based on August 2007 tallies available from CDO.

**RDs identified more than one primary work setting.

The demographic characteristics of RD survey respondents are described in Table 2.

**Table 2:** RD/Respondent characteristics

**RD/Respondent characteristics**	**N (%)**
Sex – Female	495 (97%)
Full-time practice (30+ hours/wk)	413 (74%)
Undertake public education / health promotion activities	177 (33%)
Provide one-on-one counselling services or group-level counselling/workshops	420 (77%)
	*< Monthly: 35 (8%)*
	*Monthly: 31 (7%)*
	*Weekly: 101 (24%)*
	*Daily: 257 (61%)*
**RD/Respondent characteristics**	**Average (Range)**
Age	39 (23–68)
Years in practice	15 (0–47)

### Dietitians and NHPs

RDs were asked whether they had undertaken NHP training. Table 3 presents the number and percentage of RDs who reported having undertaken training about any aspect of NHPs at the undergraduate, graduate and continuing education levels. Overall the trend is that more RDs reported undertaking training in NS than FF/N and HP. With respect to NS, undergraduate education was the most frequent source of training. For FF/N and HP, RDs turned to continuing education as their main source of training.

**Table 3:** NHP Education

**Type/Level of Education**	**Nutritional Supplements N (%)**	**Functional Foods/ Nutraceuticals N (%)**	**Herbal Preparations N (%)**
Undergraduate	341 (63%)	192 (35%)	69 (13%)
Graduate	64 (12%)	49 (9%)	25 (5%)
Continuing education	284 (53%)	264 (49%)	202 (38%)

RDs were asked whether they have ever personally ingested NHPs. 89% (142) reported having ingested NS, 82% (131) FF/N and 57% (91) HP.

RDs were asked whether NHPs are currently sold, distributed or administered at their primary employment setting. Approximately half of RDs (51% or 276) reported that NS were available at their primary employment setting, compared to only 22% (115) reporting the same for FF/N and 8% (41) for HP.

RDs were also asked whether they privately sell any of these products. Approximately 1% of RDs reported selling NS, FF/N and HP. When asked whether they currently promote or market any specific brands of NHPs on behalf of a company/store/employer, a slightly greater number of RDs reported doing so for NS (9%), FF/N (5%) and HP (1.3%).

### Demand for NHPs in dietetic services

**Figure 1:**
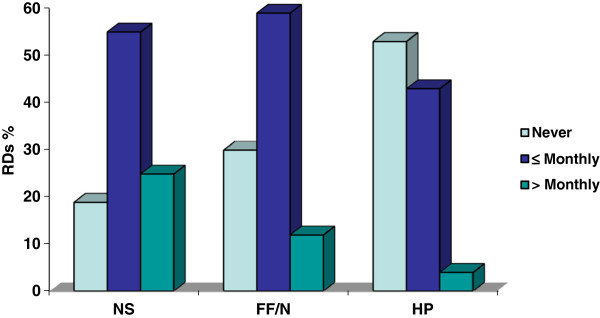
Demand for Dietetic Expertise: Colleagues Consulting with RD.

Demand for dietetic services in the area of NHPs was assessed in two ways. The first was to measure how often RDs reported that colleagues or other health care practitioners consulted with them about any aspect of NS, FF/N and HP in the past six months. These data are presented in Figure 1. The majority of RDs reported being consulted about NS (80%) and FF/N (71%), whereas just under half of RDs had been consulted about HP (47%).

**Figure 2:**
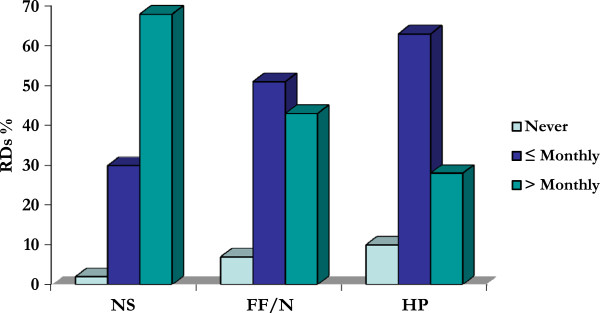
Demand for Dietetic Counselling: Questions from Clients.

The second way to measure demand for dietetic services was based on how often the subset of RDs who offer counselling services/workshops reported receiving questions from clients about any aspect of these products in the past six months. Refer to Figure 2 for these results.

The first pattern to note, in comparison with Figure 1, is that there was more demand across all products from clients than from colleagues. To turn back to Figure 2, the vast majority of RDs reported being consulted with respect to all products (98% for NS; 94% for FF/N; 91% for HP), but the overall trend is that RDs received the most frequent questions about NS and the least frequent about HP.

### Views about inclusion of NHPs in dietetic scope of practice

**Figure 3:**
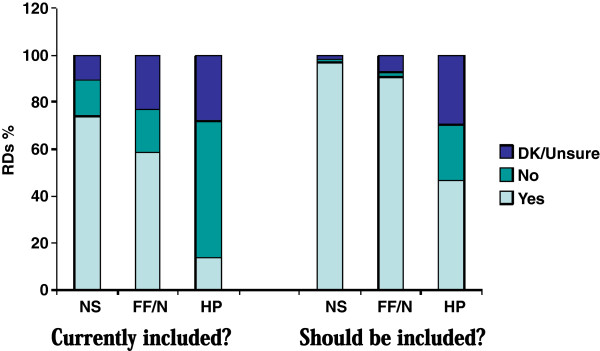
Views About Inclusion of NHP Recommendations in Scope of Practice.

This section presents data with respect to RDs’ views about whether NHPs are, or should be, within their scope of practice. Figure 3 compares the percentage of RDs who believed that the current practice of dietetics includes the capacity to make recommendations about the use of these products with the number of RDs who believed that the practice of dietetics *should* include this capacity. Substantially more RDs believed that all categories of these products *should be* included than currently are included. Specifically, 74% (381) of RDs believed that NS are included within the current scope of practice, compared to 59% (301) for FF/N and 14% (73) for HP. Even higher numbers believed that these products should be included: 97% (497) for NS, 91% (464) for FF/N and 47% (240) for HP. Overall it was clear that more RDs believed that NS, followed by FF/N and then HP should be included within their dietetic scope of practice. When two-way comparisons were made between the NS, FF/N and HP product groups, significant differences were found in all cases (p values are < 0.001 for all comparisons using the McNemar-Bowker test).

While the vast majority of RDs believed that NS and FF/N recommendations should be within their scope of practice (i.e. > 90%), there was nonetheless some variation to report with respect to views about all categories of NHPs for the following characteristics.

#### Personal consumption

Ingesting NHPs was related to expressing a belief that the recommendation of NHPs should fall within the dietetic scope of practice. Specifically, significantly more RDs who reported ingesting NS believed their scope of practice should include NS (97.9%) than RDs who had not personally ingested NS (89.2%, p < 0.01.). The same pattern holds for FF/N and HP, only it is more pronounced: more RDs who reported ingesting FF/N believed their scope of practice should include FF/N (92.4%) than RDs who had not personally ingested FF/N (75%, p < 0.001). Similarly, more RDs who reported ingesting HP believed their scope of practice should include HP (53.7%) than RDs who had not personally ingested HP (35.9%, p < 0.001).

#### NS training

RDs who had undertaken training (undergraduate, graduate or continuing education) about any aspect of NS reported in significantly higher numbers that recommending NS should be within their scope of practice. Specifically, 98.6% of RDs who had training in NS expressed this belief, compared to 91.7% of those with no NS training (p = 0.001). While a similar pattern existed for FF/N (92.1% with training compared to 87.3% with no training support inclusion), and HP (48.5% with training compared to 45.3% with no training support inclusion) these results were not statistically significant (albeit may be of practical significance).

#### Providing individual or group level counselling services/workshops relative to HP

A greater percentage of RDs who *do not* provide these services (59.7%) supported the inclusion of HP recommendations within their scope of practice than RDs who do provide these services (43%, p < 0.01). There were no significant or substantial differences in views about the inclusion of NS or FF/N.

#### Sale/distribution/administration of NS at work

RDs working in primary employment settings where NS are sold/distributed/administered were significantly more likely to believe that recommending NS should fall within their scope of practice (99.6%), compared to settings where NS are not available (94.7%, p < 0.05). There was a similar pattern with respect to FF/N, but these results were non-significant. The opposite pattern exists with respect to HPs, although these results were also non-significant.

There were no significant differences in views about the inclusion of NHP recommendations in the dietetic scope of practice with respect to whether RDs *undertake public education/promotion, promote/market NHPs,* or across *primary employment settings* (although it should be noted that in some cases the small sample size and consequently low cell counts resulted in a lack of statistical power to assess these relationships).

Uncertainty about whether these products are or should be included within the current practice of dietetics was lowest for NS and highest for HP. Specifically, 11% of RDs reported that they ‘do not know’ if NS are within the current scope of practice, compared to 23% with respect to FF/N and 28% with respect to HP. Furthermore, 2% of RDs reported that they were ‘unsure’ whether NS should be included within the current scope of practice, compared to 7% for FF/N and 30% for HP.

To control for multivariate effects, a logistic regression model was also fit for FF/N and HP, the two dependent variables for which adequate variation existed. Specifically, the following variables were regressed on views about whether FF/N or HP should be included within the dietetic scope of practice: age; undertaking public education or health promotion activities; providing one-on-one counselling services or group-level counselling/workshops; personal use of FF/N or HP; any education about FF/N or HP (undergraduate, graduate and continuing education variables were collapsed into one variable for analysis); sale/distribution/ administration of FF/N or HP at work; and promotion/marketing/private sale of FF/N or HP (promotion/marketing and private sale were collapsed into one variable for analysis). Views for FF/N and HP were recoded so that the responses ‘no’ and ‘unsure’ were grouped together, while ‘yes’ remained a separate category.

Before the full model was fit, bivariate relationships were explored. For the FF/N model, only personal use was significant, with the odds of believing FF/N should be in the dietetic scope of practice being 4.0 times higher for RDs who reported personally ingesting FF/N (p < 0.001). In the full model, again only personally ingesting FF/N was significant, with the odds ratio being 4.1 FF/N (p < 0.001).

With respect to bivariate relationships for HPs, personal use was significant, with the odds of believing HPs should be in the dietetic scope of practice being 2.1 times higher for RDs who reported personally ingesting HPs (p < 0.001). In contrast, RDs who provide one-on-one counselling services or group-level counselling/workshops were only half as likely to believe HPs should be in the dietetic scope of practice (p < 0.01). No other variables were significant. For the full HP model, the same pattern holds, with personally ingesting HP (odds ratio = 2.5, p < 0.001) and providing counseling/workshops (odds ratio = 0.52, p < 0.01) being significant.

### Limitations

With a response rate of 20%, it was important to consider whether a selection bias occurred. The majority of survey respondents were dietitians who undertake individual level counselling or provide group-level counselling/workshops. This result is consistent with the intent of the survey, which was to capture data primarily from dietitians working in these practice areas. Due to the manner in which CDO maintains its register, however, it was not possible to pre-select this targeted group of dietitians, which resulted in a decision to invite all RDs in Ontario to complete the survey. In light of this, we would consider the results generalizable primarily to dietitians providing individual or group-level counselling services or workshops.

Additionally, RDs who experienced a greater demand for their services/expertise with respect to NS and HP, and personally use FF/N were more likely to respond. This was determined by comparing results of the full survey with a secondary follow-up survey conducted several weeks later to capture data from non-responders. Although knowledge was not assessed in the follow-up survey, it is also possible that RDs with minimal or no knowledge of NHPs may not have felt comfortable responding to the survey, thus contributing to the low response rate. There were no significant differences between RDs responding to the full survey and those responding to the secondary follow-up survey with respect to primary practice setting, size of community where practicing, age, years in practice, and involvement in counselling or health promotion.

Finally, some limitations exist with the proposed set of product categories: NS, FF/N and HP. Specifically, dietitians report recommending some of the same products under more than one category (e.g. dietitians identified meal replacements under both NS and FF/N), indicating that these categories may not be perceived as wholly distinct. This is not surprising given the blurring of many products across food/medicine boundaries, in terms of intention for use by consumers and practitioners alike.

## Conclusions

The purpose of this study was to explore RDs’ perceptions about NHPs, in an effort to articulate existing or potential professional roles and responsibilities.

The findings of this study are consistent with another Canadian study with respect to growing demands for information on NHPs, and the need for increased familiarity for NS and FF/N [9].

In light of the high level of demand for dietetic services in the area of NHPs – with more than half of RDs reporting receiving questions from clients on at least a monthly basis about HP alone, and higher numbers reported for NS and FF/N -- it would appear that the *opportunity* exists for RDs to undertake a professional role with respect to NHPs. Notwithstanding the potentially overlapping roles of other health care providers in this area, RDs need to articulate their particular role. This opportunity is most relevant to RDs who undertake individual level counselling or provide group-level counselling/workshops: they were the largest group who responded to the survey and are considered the most likely to experience a greater demand for their services/expertise with respect to NHPs.

The majority of RDs believed that recommending NS was within the current dietetic scope of practice; just over half believed the same for FF/N and a minority for HP. Even more RDs believed that recommending these products *should* be within the scope of practice – particularly where NS and FF/N are concerned, and nearly half for HP. While RDs believed that NHPs should be within their scope of practice, over 20% of RDs reported that they ‘do not know’ if recommending FF/N or HP actually does fall within their scope of practice.

RDs who reported personally ingesting FF/N and HP were significantly more likely to report that these products should be in the dietetic scope of practice. In contrast, RDs who provide one-on-one counselling services or group-level counselling/workshops were significantly less likely to believe HPs should be in the dietetic scope of practice. It was not surprising that personal use, and therefore familiarity, influenced views about these products. While it was also not surprising that area of practice influences views, in light of prior research on NS and FF/N [9], it is unclear why, in particular, there was a negative relationship between counseling activities and views about HP. Unfortunately, no literature exists that explains these practice patterns.

Given current uncertainty regarding NHPs within dietetic practice, the development of a policy statement would be warranted to explicitly clarify whether specific types of NHPs fall within the dietetic scope of practice and the extent of the role of RDs with respect to these products. This recommendation does not reflect a need for a policy change per se, but rather a need for clarification of existing policy. In particular, a distinction should be made between permissible *roles* and mandated *responsibilities*, and between types of NHPs.

Since not all NHPs were perceived the same way by RDs, future work will need to continue to explore and refine the categories of NHPs that are most relevant for RDs. Further work should also assess how RDs’ respective roles and responsibilities overlap with that of other health practitioners in the area of NHPs. Clearly there are many practitioner groups who have also laid claim to (parts of) this area or who may potentially do so. Addressing this particular matter would help to clarify those instances in which RDs can or should refer clients to or consult with other health providers.

## Abbreviations

FF/N: Functional foods and nutraceuticals; HP: Herbal preparations; NHPs: Natural health products; NS: Nutritional supplements; RDs: Registered dietitians.

## Competing interests

The authors declare that they have no competing interests.

## Authors’ contributions

KH – designed and conducted the study, analyzed the data, wrote the first draft of the manuscript and approved the final version. RW – contributed to the analysis of the data and assisted with preparation of the manuscript drafts and approved the final version. HB – supervised the project and contributed feedback to the manuscript drafts. All authors read and approved the final manuscript.

## Pre-publication history

The pre-publication history for this paper can be accessed here:

http://www.biomedcentral.com/1472-6882/13/156/prepub
